# The role of IFN-γ-signalling in response to immune checkpoint blockade therapy

**DOI:** 10.1042/EBC20230001

**Published:** 2023-09-28

**Authors:** Chun Wai Wong, Yang Yu Huang, Adam Hurlstone

**Affiliations:** 1 School of Biological Sciences, Faculty of Biology, Medicine and Health, The University of Manchester, Manchester M13 9PT, U.K.; 2 Lydia Becker Institute of Immunology and Inflammation, The University of Manchester, Manchester M13 9PT, U.K.

**Keywords:** immune checkpoint inhibitor, immune evasion, immune regulation, interferon gamma

## Abstract

Treatment with immune checkpoint inhibitors, widely known as immune checkpoint blockade therapy (ICBT), is now the fourth pillar in cancer treatment, offering the chance of durable remission for patients with advanced disease. However, ICBT fails to induce objective responses in most cancer patients with still others progressing after an initial response. It is necessary, therefore, to elucidate the primary and acquired resistance mechanisms to ICBT to improve its efficacy. Here, we highlight the paradoxical role of the cytokine interferon-γ (IFN-γ) in ICBT response: on the one hand induction of IFN-γ signalling in the tumour microenvironment correlates with good ICBT response as it drives the cellular immune responses required for tumour destruction; nonetheless, IFN-γ signalling is implicated in ICBT acquired resistance. We address the negative feedback and immunoregulatory effects of IFN-γ signalling that promote immune evasion and resistance to ICBT and discuss how these can be targeted pharmacologically to restore sensitivity or circumvent resistance.

## Introduction

Inhibitory signalling in T lymphocytes (T cells), a negative feedback response to antigen stimulation, limits their ability to eradicate target cells as it renders them unresponsive to further antigen stimulation and/or weakens their effector functions. Immune checkpoint inhibitors (ICIs) are antibodies that antagonise T-cell inhibitory receptor (TCIR) signalling and thereby potentiate T-cell activation and responses, even following exhaustion from repeat tumour antigen exposure. ICI targeting cytotoxic T-lymphocyte-antigen 4 (CTLA-4), programmed cell death 1 (PD-1) and more recently lymphocyte activation gene-3 (LAG-3) have entered the clinic as monotherapies but work even more potently in combination, transforming outcomes for cancer patients, especially for those whose tumours carry a high mutational burden and are T-cell-inflamed [1–4]. IFN-γ is a cytokine that activates cellular immune responses, and its production is linked to immunosurveillance of cancer cells and clinical responses to immune checkpoint blockade therapy (ICBT). However, there is a dark side to IFN-γ wherein it promotes immune evasion and resistance to ICBT [5–8]. Potentially, selectively antagonising aspects of IFN-γ signalling associated with feedback inhibition or its role in immunosuppression could restore sensitivity to or circumvent resistance to ICBT.

## An overview of IFN-γ signalling

**Figure 1:**
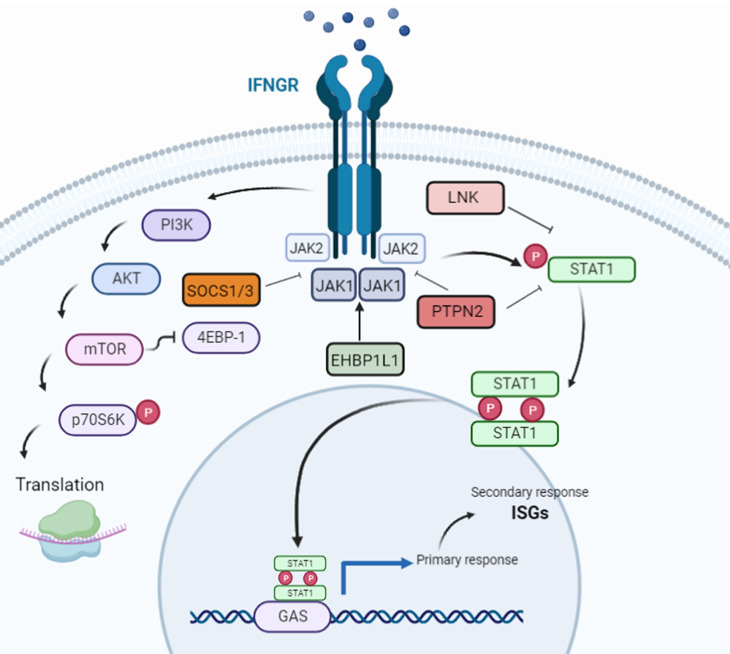
An overview *of* the IFN-γ signalling pathway The IFN-γ receptor (IFNGR), a heterodimer of IFNGR1 and IFNGR2, is expressed on the surface of almost all cell types. After IFN-γ binds to IFNGR, it induces the activation of Janus kinase (JAK) 1 and JAK2 while EHBP1L1 is responsible for stabilising JAK1. These then phosphorylate tyrosine 701 (Try701) of signal transducer and activator of transcription (STAT) 1 to induce homodimer formation. STAT1 dimers enter the nucleus and bind to the IFN-γ activated sites (GASs) of target genes to initiate their transcription. A principal target is the transcription factor IRF1, which activates expression of many interferon-stimulated genes (ISGs). Additionally, IFN-γ stimulates the phosphoinositide-3-kinase–protein kinase B/Akt (PI3K-Akt) pathway, which is also required for the full activation of STAT1 in the canonical pathway. Suppressor of cytokine signalling 1/3 (SOCS1/3), LNK, and PTPN2 are responsible for acting as feedback inhibitors, deactivating JAK1/2, to maintain homeostasis of IFN-γ signalling (Created with BioRender.com).

IFN-γ is an ∼50 kDa homodimer formed by non-covalent antiparallel association of two 17 kDa polypeptide subunits with multiple N-glycosylations [9,10] secreted by T cells, natural killer (NK) cells, invariant NKT cells (iNKT cells), regulatory T cells (Tregs), γδ (γδ) T cells and B cells in response to interleukin (IL) -12, -15, -18, and -21 [11,12] and T-cell receptor (TCR) signalling, mediated by transcription factors Activator protein 1 (AP-1), T-box transcription factor TBX21 (T-bet), Eomesodermin (EOMES), nuclear factor of activated T cells (NFAT), and nuclear factor kappa B (NF-κB) [13–15]. IFN-γ signalling has been extensively reviewed elsewhere [5] and is outlined in Figure 1.

## Anti-proliferative and pro-apoptotic effects of IFN-γ

IFN-γ is a tumour suppressor which exerts direct anti-proliferative effects on a variety of tumour cells through effects on the cell cycle [16]. The gene encoding cyclin-dependent kinase (CDK) inhibitor p21^WAF1/CIP1^ is a STAT1 target whose product inhibits entry into and progression through the cell cycle [17]. IFN-γ is also a cytotoxic cytokine that induces p53 stabilisation and stimulates apoptosis through both the intrinsic and extrinsic pathways, increasing expression of BCL2 associated agonist of cell death (Bad), BH3 interacting-domain death agonist (Bid), Bcl-2 homologous antagonist/killer (Bak), Fas Cell Surface Death Receptor (Fas) and Fas ligand (FasL), cytochrome *c* release from mitochondria, and activating caspases [18–20]. IFN-γ activates caspase 8 and caspase 3, triggering apoptosis in colorectal cancer cell lines [21]. In breast and lung cancer cells, IFN-γ enhances the expression of caspase 8, increasing sensitivity to apoptosis elicited by FasL/tumour necrosis factor (TNF)-related apoptosis-inducing ligand (TRAIL) [22–25]. Furthermore, IFN-γ mediates its tumouricidal effect through inducing necroptosis dependent on activation of the serine-threonine kinase receptor-interacting protein kinase 1 (RIPK1) [26].

## Anti-angiogenic effect of IFN-γ

IFN-γ inhibits angiogenesis, thereby inducing tumour ischemia, by impairing endothelial cell proliferation and survival. IFN-γ directly inhibits the proliferation of vascular endothelial cells [27–29]. IFN-γ also inhibits the production of vascular endothelial growth factor (VEGF) required for endothelial cell proliferation, survival, and migration, and promotes the production of anti-angiogenic substances. IFN-γ switches M2-polarised tumour-associated macrophages (TAMs), the main sources of VEGF in the tumour microenvironment (TME), to an M1 phenotype, reducing VEGF production [30,31]. In addition, IFN-γ produced by T helper 1 (Th1) cells makes macrophages directly cytotoxic to cancer cells, and causes them to secrete angiostatic chemokine ligand 9 (CXCL9) and CXCL10 to inhibit angiogenesis [32].

## IFN-γ-driven anti-tumour cellular immune responses

The immune stimulatory effects of IFN-γ have been reviewed extensively [5] and can be summarised as follows: IFN-γ enhances antigen processing and presentation in transformed cells as well as in professional antigen presenting cells and stimulates the recruitment, proliferation and polarisation of several leukocytes that promote or mediate clearance of transformed cells, including dendritic cells, macrophages, T helper cells, and cytotoxic lymphocytes. As ICBT requires cellular immune responses to be effective, IFN-γ signalling has emerged as a strong correlate of clinical response [33,34]. Data collated and analysed from more than 1000 patients with different types of cancers treated with ICBT showed that intratumoural CXCL9 expression strongly correlated with ICBT response [35]. CXCL9 is an IFN-γ target gene essential for recruiting T cells to tumour sites for immune clearance [36,37]. Transcriptomic analysis of ∼400 NSCLC patients treated with ICBT corroborated that high IFN-γ activity correlated with response, and, moreover, that the IFN-γ-induced immunoproteasome was essential [38]. Interestingly, collating data from seven different cohorts of melanoma patients treated with ICBT revealed that patients treated firstly with anti-CTLA4 followed by anti-PD-1 who displayed high IFN-γ signalling in tumour biopsies responded best to ICBT [39].

Further underscoring the requirement for IFN-γ signalling for ICBT response, multiple studies have reported that mutations which impair IFN-γ signalling in tumour cells promote ICBT resistance [40–49] (see also Table 1). Melanoma with intrinsic loss of IFN-γ signalling possess fewer tumour infiltrating lymphocytes (TILs) with impaired function [50]. Additionally, nonsense mutations in the Apelin Receptor (APLNR), which interacts with JAK1 to strengthen the IFN-γ response, were detected in patients with advanced melanoma or lung cancer resistant to ICBT; restoration of APLNR improved survival of mice with APLNR-defective melanomas treated with ICBT by increasing the responsiveness of tumour blood vessels to IFN-γ and augmenting the effectiveness of anti-tumour T cells [51]. However, mutations which impair IFN-γ signalling in cancer even with resistance to ICBT are relatively rare ([52–54] and Table 1). Moreover, using meta-analysis, Song and colleagues concluded that the loss of IFN-γ signalling sensitises tumour cells to immune effector cells *in vitro*, made tumours more susceptible to the host immune system *in vivo*, and patient tumours developing mutations in IFN-γ signalling before treatment were more likely to respond to ICBT [55]. Further, interferon-stimulated gene products were enriched in serum from non-responders with metastatic melanoma treated with ICBT [56]. Indeed, several studies have now established that IFN-γ signalling can result in ICBT resistance together with approaches to tackle this, the subject of the remainder of this review.

**Table 1:** A summary of the literature addressing the relationship between IFN-γ signalling and the response of cancer patients to ICBT (those supporting a positive role are bold; those a negative role are not)

Major finding related to IFNG	Reference
Patients identified as non-responders to anti-CTLA-4 (ipilimumab) have tumors with genomic defects in IFN-γ pathway genes	[40]
Resistance-associated loss-of-function mutations in genes encoding JAK1 or JAK2 concurrent with deletion of the wild-type allele in patients with metastatic melanoma who had an initial objective tumor regression in response to anti–PD-1	[43]
**Genes including GZMA, PRF1, PD-L2, and CTLA4, CD8A/B, PD-L1, LAG3, and IFNG not more highly expressed in anti-PD-1-responsive melanoma**	[52]
IFN-γ-responsive genes appear necessary, but not always sufficient, for clinical benefit from different clinical studies of anti-PD1 ICBT	[33]
**Neither JAK1 and JAK2 mutations nor copy-number alterations in IFN-γ pathway genes associated with ICBT resistance in this cohort**	[49]
**IFN-γ pathway genes not significantly different with regard to somatic point mutations or indels between responders and nonresponders with melanoma treated with sequential anti-CTLA-4 and then anti-PD-1**	[54]
B2M aberrations in 29% of metastatic melanoma patients treated with ICBT with progressing disease, including multiple frameshift mutations. LOH overlapping B2M, and absence of tumor-specific B2M protein expression. Association of LOH overlapping IFNGR1 with poorer overall survival exclusively in the anti-PD1 cohort. Six JAK1 alterations, including a missense mutation in one non-responder, and LOH in five more patients (two non-responders, two resistant patients, and one responder). LOH overlapping JAK2 detected in 10 samples (4 non-responders, 3 resistance patients, and 3 responders). LOH overlapping IFNGR1 detected in 5 samples (3 non-responders, 1 resistant patient, and 1 responder).	[47]
IFNGR1 mutations were found only in responders with non-small-cell lung cancer (NSCLC) treated with combination anti-PD-1/anti-CTLA-4	[45]
IFNGR1 expression significantly higher in non-responders with metastatic urothelial cancer treated with anti-PD-L1. IFNγ expression correlates with CD8+ Teff activity.	[48]
IFN-γ response enriched in responders with melanoma treated with anti-PD1	[46]
IFN-γ response significantly enriched in responders in the anti-CTLA4-treated subgroup	[46]
T cell infiltration and IFN-γ signaling signatures correlate with clinical response to therapy	[34]
Intratumoural expression of CXCL9 strongly correlates with the likelihood of responding to ICBT in more than 1000 cancer patients	[35]
**High IFN signalling with NK response priming predicted associated with poor survival, while T cell response priming predicted associated with a favourable response (datasets combined from Liu, Gide, and Riaz papers). ISG expression was associated with improved survival in tumors with 6p21.3 disomy**. **Serum IFNγ and HLA-E expression in non-responders at 6 months posttreatment from 202 ICBT-treated individuals with melanoma.**	[56]
**Melanoma patients progressing on ICBT exhibit loss of antigen expression related to high tumour-intrinsic IFN-γ signaling**	[52]
**Meta-analyses confirms that loss of IFN-γ signalling in tumour cells caused resistance to immune effector cells *in vitro*, made tumours more susceptible to the host immune system *in vivo*, and patient tumours developing mutations in IFN-γ signalling before treatment were more likely to respond to ICBT**	[55]
Intratumoural expression of IFN-induced immunoproteasome strongly correlates with the likelihood of responding to ICBT in close to 400 NSCLC patients	[38]
Melanoma patients firstly treated with anti-CTLA4 followed by anti-PD-1 therapy who induce a high IFN-γ signalling in tumour biopsies have best response to ICBT treatment	[39]

## How does IFN-γ signalling contribute to immune evasion and resistance to immune checkpoint blockade therapy?

**Figure 2:**
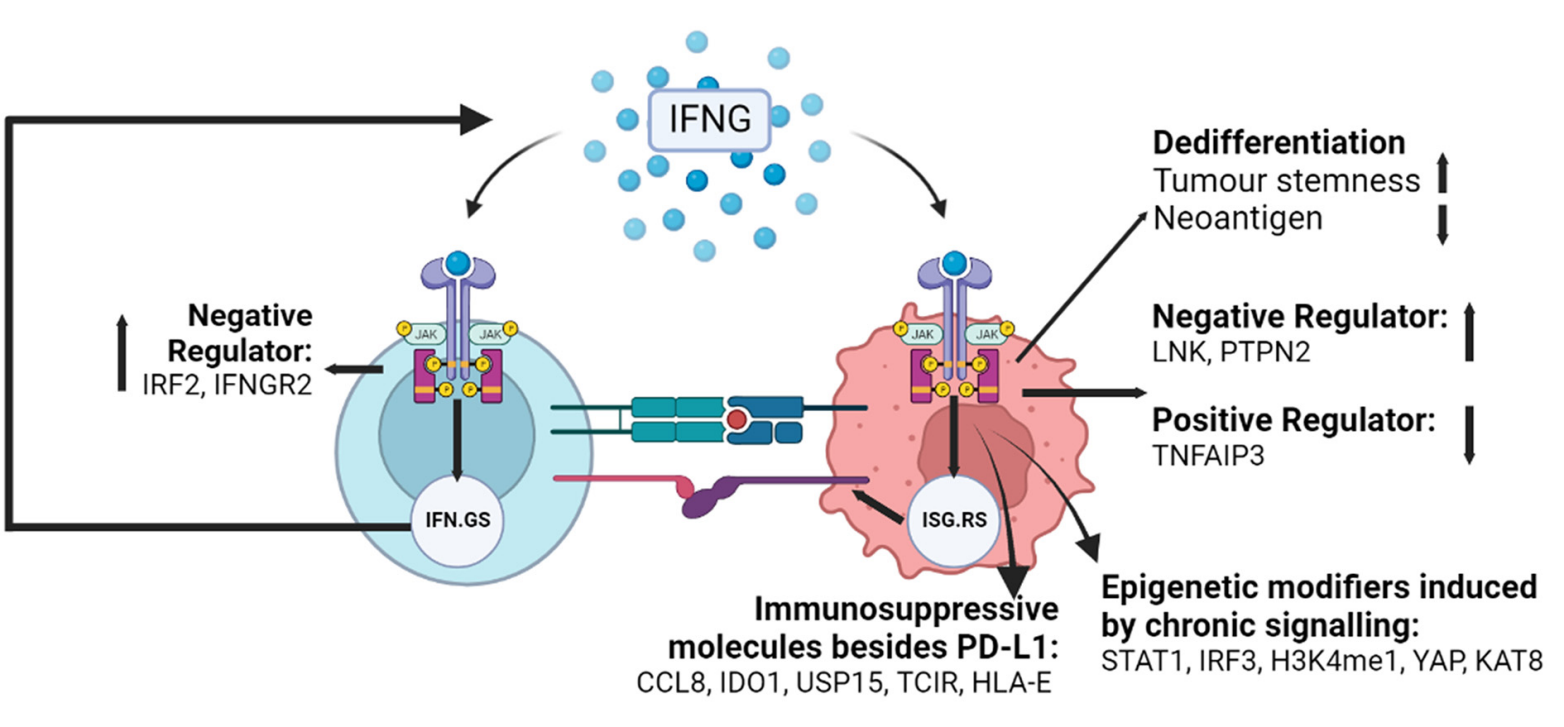
Different mechanisms elicited by IFN-γ to promote resistance to ICBT IFN-γ drives tumour cell dedifferentiation by either up-regulating its stemness or downregulating its neoantigen expression. IFN-γ feedback loops become dysregulated, by either overexpressing the negative regulators LNK and PTPN2, or downregulating the positive regulator TNFAIP3. Chronic IFN-γ signalling in tumour cells induces epigenetic modifiers STAT1, IRF3, H3K4me1, YAP, and KAT8 to induce interferon stimulated genes resistance signature (ISG.RS) that encode several immunosuppressive molecules including PD-L1, CCL8, IDO1, USP15, TCIR, and HLA-E. In contrast, interferon gamma hallmark geneset (IFN.GS) is induced by IFN-γ in T cells to augment anti-tumour responses but this gene program is inhibited by the negative regulators of IFN-γ signalling IRF2 and IFNGR2 (Created with BioRender.com.).

To date, IFN-γ-driven immune evasion and ICBT resistance mechanisms are mainly explained by (1) the induction of immunosuppressive molecules, (2) the up-regulation of negative-feedback regulators, (3) epigenetic effects of chronic exposure, and (4) driving dedifferentation of cancer cells or otherwise affecting antigen processing (Figure 2).

## Induction of immunosuppressive molecules

IFN-γ can promote immunosuppressive effects through increasing PD-L1 and indoleamine 2, 3-dioxygenase 1 (IDO1) expression in tumours [5]. PD-L1 is a ligand binding the PD-1 receptor on T cells resulting in their exhaustion [57]. Further, PD-L1 expression lowers tumour cell susceptibility to killing by NK cells [58]. Ubiquitin specific peptidase 15 (USP15) is a deubiquitinase which inhibits the the generation of IFN-γ-producing Th1 cells [59]. Mice with USP15-deficient T cells were found to generate excessive IFN-γ, which up-regulated expression of PD-L1 and CXCL12, causing effector T-cell exhaustion alongside infiltration of Tregs and myeloid-derived suppressor cells (MDSCs) into tumours, which promoted tumour formation [59]. IDO1 is an enzyme implicated in degradation of tryptophan, needed by T cells for activation, and simultaneously generating kynurenine, a metabolite capable of dampening immune responses. IDO1 activity can also induce the generation of Tregs to inhibit the killing function of cytotoxic lymphocytes (CTLs) [60–62]. Furthermore, IDO1+ DCs generated by IFN-γ inhibit allogeneic T-cell responses despite simultaneously secreting pro-inflammatory cytokines [63,64].

Interestingly, IFN-γ driven PD-L1 and IDO1 up-regulation became the dominant pathway inducing resistance to ICBT following TGF-β blockade in a mouse lung adenocarcinoma model [65]. While simultaneous TGF-β and PD-L1 blockade in this model resulted in significantly improved survival compared with anti-PD-L1 therapy alone, prolonged combination treatment promoted resistance by up-regulating PD-L1 and IDO1 through increased IFN-γ signalling [65]. However, tumour relapse could be prevented by JAK inhibition [65].

Besides PD-L1 and IDO1, there are several other immunosuppressive chemokines being upregulated by IFN-γ to elicit immune evasion. Ultraviolet radiation induces macrophages to secrete IFN-γ, which in turn induces melanocytes to secrete chemokine (C-C motif) ligand 8 (CCL8) that promotes inflammation and immune evasion [66]. In addition, an intrinsic high activity of IFN-γ signalling in tumour cells shapes an immunosuppressive TME. Several short-term melanoma cell cultures generated from patients who progressed on ICBT exhibited high intrinsic IFN-γ signalling activity while the corresponding tumour biopsies demonstrated low abundance of activated CD8+ effector T cells [52]. Moreover, IFN-γ was found to be responsible for promoting the expression of histocompatibility leukocyte antigen chain E (HLA-E) in tumour cells, which inhibits the anti-tumour responses of CD8+ T cells and NK cells [56].

## Negative-feedback regulators of IFN-γ signalling

Different negative regulators of IFN-γ signalling can be up-regulated in tumours in response to IFN-γ to induce immune evasion. Lymphocyte adapter protein (LNK) was up-regulated in melanoma and LNK genetic ablation restored IFN-γ signalling and a significant reduction of tumour size in different preclinical tumour models [67]. IFN-γ signalling was inhibited by protein tyrosine phosphatase non-receptor type 2 (PTPN2) whose deletion in tumour cells increased ICBT efficacy by enhancing IFN-γ-mediated effects on antigen presentation and cell growth inhibition [68]. N-acetylglucosaminyltransferase III (MGAT3) destabilises IFN-γ receptor α chain (IFN-γR1) through N-glycosylation and proteasome-dependent degradation also contributes to ICBT resistance [69]. IFN-γ-treated cancer cells also upregulate RIPK1, which connects TNF signalling to NF-κB that in turn induces an immunosuppressive chemokine program [70]. Deleting RIPK1 in tumour cells lowered infiltration by immunosuppressive macrophages in tumours, promoting TNF-induced killing and improving ICBT response [70]. Negative regulators of IFN-γ-signalling in immune cells also contribute to ICBT resistance. IRF2 induced by IFN-γ-in T cells results in exhaustion in multiple tumour types [71]. Deletion of IRF2 in CD8+ T cells increased effector functions to sustain long-term tumour control in response to ICBT [71]. IFN-γ receptor β chain (IFN-γR2) in CD8+ T cells also restricts anti-tumour responses and again selective deletion of IFN-γR2 reinvigorated stem-like T cells, strengthening anti-tumour immunity [72].

Although many reports have suggested that antagonising negative regulators of IFN-γ signalling reverses resistance to ICBT, other reports suggest the opposite rationale to overcome resistance. Tumour cell intrinsic loss of TNFα-induced protein 3 (TNFAIP3) promotes lung tumourigenesis associated with reduced CD8+ T cell–mediated immunosurveillance in patients and in mouse models [22]. The effects of TNFAIP3 loss were largely explained by an increased cellular sensitivity to IFN-γ signalling by the aberrant activation of TANK-binding kinase 1 (TBK1) and STAT1. Deleting the IFN-α/β (IFN-α/β) receptor restored cytotoxic T cell infiltration into tumours, thereby antagonising tumourigenesis [22]. Furthermore, IFN-γ signalling can be effectively maintained within tumours through EH domain-binding protein 1-like protein 1 (EHBP1L1) shielding JAK1 from proteasomal degradation. This mechanism promoted PD-L1 expression in a mouse model of renal cell carcinoma (RCC) and EHBP1L1 inhibition resulted in increased CTL activity and improved response to ICBT [73].

## Chronic exposure to IFN-γ in tumours and tumour draining lymph nodes

Chronic exposure of tumours to IFN-γ induces resistance to ICBT through epigenetic and transcriptional changes. Chronic-IFN-γ-treatment of tumour cells induced STAT1-associated epigenomic changes, promoting IFN-γ-independent expression of ISGs including ligands for multiple TCIRs, such as TNF receptor superfamily member 14 (TNFRSF-14) and MHC-II, that conferred resistance to ICBT in preclinical models [74]. Moreover, resistance to ICBT could arise in both antigen-sufficient and insufficient tumours through the up-regulation of T-cell terminal exhaustion in the former and increased immaturity of innate immune cells in the latter [75]. STAT1 is not the only transcription factor responsible for the transcriptomic and epigenomic changes in tumours elicited by chronic IFN-γ exposure. IRF3 and associated histone 3 lysine 4 mono-methylation (H3K4me1) enhance the chromatin accessibility and increase expression of several key immune-inhibitory IFN-stimulated genes [76]. Furthermore, IFN-γ promotes nuclear translocation and phase separation of Yes-associated protein (YAP) in tumour cells, which mediated adaptive resistance to ICBT by forming transcriptional hubs with TEA domain transcription factor 4 (TEAD4), E1A-associated protein p300 (EP300), and mediator of RNA polymerase II transcription subunit 1 (MED1) to maximise cancer-promoting gene transcription [77]. Additionally, IFN-γ induces expression of histone K (lysine) acetyltransferase 8 (KAT8), resulting in its interaction with IRF1, thereby promoting IRF1-induced chromatin acetylation and hence upregulation of PD-L1 expression in tumour cells, contributing to immune evasion [78].

Besides IFN-γ in tumours playing a major role in promoting resistance to ICBT, recent findings support that IFN-γ outside tumours also influences resistance to ICBT, especially in tumour-draining lymph nodes. Chronic IFN signalling induced epigenetic rewiring in tumour cells, which up-regulated expression of PD-L1 and MHC-I to suppress NK and T cells activities, thereby promoting lymph node colonisation. Within lymph nodes, tumour cells chronically exposed to IFN-γ induce Treg formation that then promote distant metastasis [79]. It has also been shown that lung-associated lymph nodes abundant in IFN-γ promote the development of immunosuppressive Th1-like Tregs, which interact with type 1 conventional dendritic cells and thereby limit the priming of CTL responses against lung cancer [80].

## Dedifferentiation of cancer cells and altered antigen processing

The panoply of antigens expressed by tumour cells and potentially presented to the immune system depends on their differentiation status [81]. Reprogramming of gene expression by IFN-γ can radically alter antigen expression as well as conferring other cellular traits contributing to cancer progression. IFN-γ induced interferon-induced protein with tetratricopeptide repeats 5 (IFIT5), C-X-C Motif Chemokine Receptor 4 (CXCR4) and branched-chain amino acid aminotransaminase 1 (BCAT1) all promote cancer cell stemness and metastasis [82–84]. Moreover, two further reports found that low-dose IFN-γ promoted tumour colonisation of lung tissues [85,86]. Further, which peptides end up being presented on major histocompatibility complex (MHC) class I depends in large part on how antigens are degraded by the proteasome [87]. Previously, two research groups reported that IFN-γ lowered neoantigen expression by inducing an immune proteasome, which caused their degradation in a way that prevented loading on MHCI [88,89]. In addition, IFN-γ can mask the recognition of tumour cells by CD8+ T cells through increasing expression of non-cognate MHC-I molecules in tumours such as Ova_257_/H-2Kb complexes [90]. Cho and colleagues also mentioned a similar phenomenon and further explored inhibiting IFN-γ signalling to optimise peptide vaccine efficacy [90].

## Current approaches to tackle IFN-γ-driven resistance to ICBT

**Figure 3:**
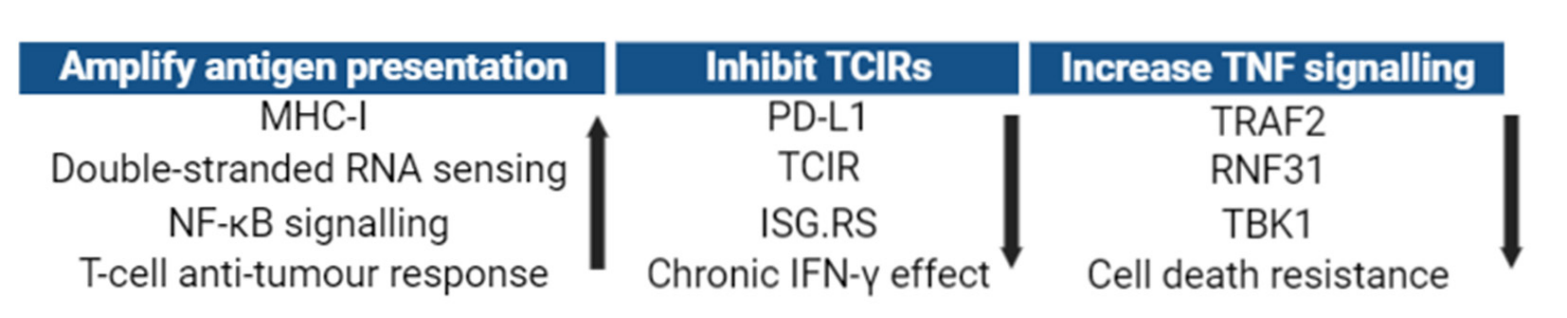
Current pharmacological approaches to reverse IFN-γ-driven resistance to ICBT Amplifying antigen presentation can be applied to upregulate tumour MHC-I, double-stranded RNA sensing, and NF-κB signalling to augment the T-cell anti-tumour response. Inhibiting TCIRs by JAK1/JAK2 inhibitor (JAKi) can decrease expression of immunosuppressive molecules, including but not limited to PD-L1 and other TCIR. Increasing TNF signalling by lowering TRAF2, RNF31, or TBK1, which are all negative regulators, will downregulate the immunosuppressive effect of tumour IFN-γ signalling and thereby reinvigorate the anti-tumour response (Created with BioRender.com.).

In general, there are three main directions to tackle this challenge therapeutically, which are (1) amplifying tumour antigen presentation, (2) inhibiting the action of T-cell inhibitory receptors, or (3) increasing tumour necrosis factor (TNF) signalling in tumours (Figure 3). However, many key regulators are generally not druggable, or are critical for host survival, or the clinical effect is not as potent as in preclinical models. Therefore, targeting IFN-γ-driven resistance to ICBT remains a significant hurdle. For instance, genetic deletion of RIPK1 in mice causes embryonic lethality [91]. In addition, inactivating the kinase activity of RIPK1 in cancer cells conferred minimal effects on preventing suppressive chemokine production and did not enhance TNF-mediated cytotoxicity [70]. In a large phase 3 trial (ECHO-301/KEYNOTE-252), IDO1 inhibitor Epacadostat co-administered with Pembrolizumab (a clinically approved PD1-targeting ICI) failed to improve progression-free survival compared with Pembrolizumab alone [92]. IDO1-inhibiton even protected tumour cells from switching off protein translation and Melanocyte Inducing Transcription Factor (MITF) production by IFN-γ [93].

To amplify antigen presentation in tumours, different researchers suggest using various immunomodulatory substances. BO-112, a nanoplexed version of polyinosinic:polycytidylic acid (poly I:C), activates double-stranded RNA (dsRNA) sensing (via protein kinase R and Toll-like receptor 3) and induces MHC class I expression via NF-κB signalling to restore the activation of tumour-specific T cells [94]. BO-112 was tested in a clinical trial (NCT04570332) to examine its efficacy when combined with Pembrolizumab in patients with advanced and/or metastatic melanoma that have progressed on Pembrolizumab alone treatment. However, the clinical trial results have not yet been reported. Paradoxically, up-regulating MHCI expression can despite making cancer cells more visible to T cells down-regulate immunosurveillance. Taniguchi and colleagues showed that the up-regulation of MHCI molecules on tumour cells after IFN-γ treatment resulted in a decrease in NK cell sensitivity and enhanced lung colonisation and metastasis of melanoma [95]. Takeda and colleagues also observed that breast adenocarcinoma cells overexpressing IFN-γ increased their resistance to NK cell attack [96].

JAK1/JAK2 inhibitor (JAKi) has been applied to inhibit expression of TCIR ligands and improved ICBT response in preclinical tumour models [74]. In addition, JAKi was reported to decrease tumour stemness, tumour-promoting chemokines and cytokines, and immunosuppressive MDSCs [97,98]. Currently, JAKi is being clinically evaluated in patients with advanced solid tumours (NCT02646748), non-small cell lung cancer (NCT02917993), and triple-negative breast cancer (NCT02876302). A trial (NCT03425006) that utilised sequential JAKi and anti-PD-L1 antibodies revealed enhanced clinical responses in patients with metastatic non-small cell lung cancer, particularly when administered after discontinuing treatment with anti-PD-1 antibodies [99]. The researchers also observed that JAKi promoted increased CD8 T-cell plasticity and enhanced therapeutic responses in exhausted and effector-memory clonotypes [99]. However, concerns about JAKi safety have been raised due to its myelosuppressive effects in humans [100].

TNF is present in lower abundance in tumours in non-responders to ICBT compared with responders [101]. Consequently, reducing the threshold to TNF-induced cytotoxicity in tumours could enable T cells to effectively eliminate tumour cells even in cancer patients with low TNF abundance [101]. Receptor-associated factor 2 (TRAF2) was identified as a gatekeeper of TNF cytotoxicity threshold in tumours, by suppressing RIPK1-dependent cell death [101]. Applying a second mitochondria-derived activator of caspase (SMAC) mimetic degrading both Baculoviral IAP repeat-containing protein 2 (BIRC2) and BIRC3, which target the TRAF2 interaction partner cellular inhibitor of apoptosis protein 1 (cIAP1), restored sensitivity to ICBT in several preclinical tumour models [101]. Addtionally, inhibition of E3 Ubiquitin-Protein Ligase RNF31 disrupted the cell ligand-bound TNF receptor complex I, which resulted in TNFAIP3 and inhibitor of NF-κB kinase (IKK) complex loss. This sensitised tumour cells with MHC/antigen-deficiency which could not be killed by immune cells directly and synergised with ICBT [102]. Another way to lower the threshold to TNF cytotoxicity that was recently suggested is to target the innate immune TANK-binding kinase 1 (TBK1). TNF- or IFN-γ-treated TBK1-null tumour cells promoted RIPK- and caspase-dependent cell death regulators [103]. Interestingly, TBK1-loss did not slow down tumour growth in immunodeficient mice; however, targeting TBK1 pharmacologically promoted a more immunostimulatory effect in the TME because it increased the infiltration of CD8 T cells and M1 macrophage into tumours [103]. Also, TBK-inhibitor treatment of CD8 T cells and M1 macrophages increased their expression of effector cytokines, including TNF and IFN-γ [103].

## Summary

- The importance of the field: IFN-γ is a pleiotropic cytokine with pro- and anti-tumour effects. However, following recent breakthroughs, how it drives resistance to ICBT is becoming clearer.
- A summary of the current thinking: IFN-γ-driven resistance to ICBT is associated with its low dose, induction of negative regulators, chronic exposure, and overexpression of immunosuppressive molecules in tumours.
- Comments on future directions: To tackle this mode of resistance, researchers should explore ways to identify novel positive or negative regulators of IFN-γ signalling in tumours, which could be pharmacologically modulated.

## Open Access

Open access for this article was enabled by the participation of University of Manchester in an all-inclusive *Read & Publish* agreement with Portland Press and the Biochemical Society under a transformative agreement with JISC.

## Abbreviations

BCAT1: branched-chain amino acid aminotransaminase 1
cIAP1: cellular inhibitor of apoptosis protein 1
CTLA-4: cytotoxic T-lymphocyte-antigen 4
dsRNA: double-stranded RNA
HLA-E: histocompatibility leukocyte antigen chain E
ICBT: immune checkpoint blockade therapy
ICI: immune checkpoint inhibitor
IFN-γ: interferon-γ
LAG-3: lymphocyte activation gene-3
MDSC: myeloid-derived suppressor cell
MHC: major histocompatibility complex
PD-1: programmed cell death 1
RCC: renal cell carcinoma
TCIR: T-cell inhibitory receptor
TNF: tumour necrosis factor
